# A generative model for scientific concept hierarchies

**DOI:** 10.1371/journal.pone.0193331

**Published:** 2018-02-23

**Authors:** Srayan Datta, Eytan Adar

**Affiliations:** 1 Department of Computer Science and Engineering, University of Michigan, Ann Arbor, MI, United States of America; 2 School of Information, University of Michigan, Ann Arbor, MI, United States of America; University of Oxford, UNITED KINGDOM

## Abstract

In many scientific disciplines, each new ‘product’ of research (method, finding, artifact, etc.) is often built upon previous findings–leading to extension and branching of scientific concepts over time. We aim to understand the evolution of scientific concepts by placing them in phylogenetic hierarchies where scientific keyphrases from a large, longitudinal academic corpora are used as a proxy of scientific concepts. These hierarchies exhibit various important properties, including power-law degree distribution, power-law component size distribution, existence of a giant component and less probability of extending an older concept. We present a generative model based on *preferential attachment* to simulate the graphical and temporal properties of these hierarchies which helps us understand the underlying process behind scientific concept evolution and may be useful in simulating and predicting scientific evolution.

## Introduction

In numerous domains, science work produces specific ‘artifacts,’ including models, methods, theories, algorithms, systems. (We distinguish, though only nominally, between *produced* (i.e., through construction) and *discovered*. A discovery may include the identification of new natural ‘entities’ (e.g., organisms, genes, planets).) In most situations, produced artifacts–or *concepts* when they are named– connect to others: one model may build on another; a new theory may refine or compete with an existing one, and one algorithm may extend another. We can model this ‘progression’ as a dynamic graph, where nodes, representing artifacts are added over time. Edges, similarly dynamic, describe an Extends relationship between two artifacts (or inversely, ExtendedBy). For example, *Least-Squares Support Vector Machines (LSSVM)* Extends *Support Vector Machines (SVM)* and is itself ExtendedBy *Weighted Least-Squares Twin Support Vector Machines (WLSSVM)*. Taken together these form three nodes in our broader graph.

Attempts to capture and describe the generative process behind this structure have largely focused on the use of citation networks. Insofar as conference and journal articles describe the artifacts, and citation denotes ‘extension,’ these networks have been modeled both in static and dynamic states. In this type of analysis, documents serve as proxies for scientific work product and citations as an indicator of extension [1–3]. However, to model scientific concept evolution this approach is limited. First, documents are not always focused on a single concept. Second, perhaps more critically, the purpose of citation is wide, varied and ambiguous. Because of this, citations are poor proxies capturing the notion of extension [4]. A citation network is at best a weak representation (and modeling instrument) for the underlying progression of scientific concepts.

We instead pursue a content-based approach and use scientific keyphrases instead of documents as fundamental units. These scientific keyphrases are mined from academic corpora. In prior work, we developed a mechanism for extracting concept keywords from documents using keywords [5]. Using naming conventions, we described an effective mechanism to find likely concept extensions.

Qualitatively, we broadly classify concepts into two categories: concepts that are entirely new or concepts that build upon previously established concepts (these are analogous to *jump* and *derived* [6]). Concepts that build upon multiple other concepts can act as ‘bridges,’ and link different sub-domains of scientific research. For example, *Single Instruction Multiple Thread Evolution Strategy Pattern Search* connects three different scientific sub-domains—*Evolution(ary) Strategy*, *Pattern Search*, and *Single-Instruction-Multiple-Thread*. A link in our hierarchy is created from a derived concept to the concept it extends. We build a hierarchical network structure (i.e., directed acyclic graph) using a set of concepts, where an edge from one concept to another denotes that the first concept extends the other one. A concept’s ‘birthdate’ (the first time it appeared) allows us to study the hierarchy over time. As with temporal data on author and publication based models [7], leveraging concept hierarchies can provide useful information about emergence in a scientific field.

In this study, we limit our experiment to a specific scientific discipline, Computer Science (CS). With computer science, we have significant historical coverage of the literature in full text. An analysis of properties of the concept hierarchies of CS reveals power-law degree distribution (some concepts are much more likely to be extended compared to others), a power-law component size distribution or existence of a giant component. Unfortunately, many existing generative models for graphs with power-law degree distribution do not focus on any other properties of the graph. These models often assume that there is a single connected component in the graph, which is not the case with scientific concept hierarchies. Simple generative models also make it difficult to model the dynamics of the hierarchies as the temporal properties are not related to graph structure.

We propose a generative model based on an extended *preferential attachment* model and other empirical observations to simulate hierarchies with similar properties.

We contribute an empirical study the graphical and temporal properties of phylogenetic hierarchies of scientific concepts. We demonstrate that starting from very simple intuitions (e.g., ‘current concept popularity predicts future extension’ and ‘older concepts are less likely to be extended compared to newer concepts’), our model produces a very good match for the actual hierarchies. We measure the fit by comparing key properties such as degree distribution, component size distribution, the diameter of each component and temporal properties not based on graph structure.

## Related work

Past work on modelling scientific evolution has focused on studying relations between scientific documents [2, 3] through citation data analysis [1]. Our content-centric approach aims to provide a generative model for the concept hierarchies, and hence we focus on related work on modeling networks.

Generative models for graphs are commonly applied to modelling real-world interactions with Erdős-Rényi random graph models being some of the first [8]. With the growth of available network data (biological, social, text, etc.) saw an emergence of new and extended generative models. Albert et al. [9] and Broder et al. [10] showed that the in and out-degree distribution of the World Wide Web (WWW) follows a power-law degree distribution. Faloutsos et al. [11] showed that the Internet topology also follows power-law degree distribution. A power-law distribution of citations in scientific literature was originally due to observations by Lotka [12]. Many other networks including movie actor collaboration network [13], scientific collaboration network [14], the network of human sexual contact [15] are all shown to follow power-law (scale-free) degree distributions.

Several generative models have been devised to explain the formation of these structures. The most well-known among these are Barabási-Albert model [13] (used for WWW and scale-free social networks), and the similar, Price’s model [16] (used in modelling citation networks). Both models rely on *preferential attachment* or the ‘rich gets richer’ phenomenon. Preferential attachment processes are a set of stochastic processes which assigns ‘wealth’ to a set of individuals based on the ‘wealth’ they currently have. The first use of these processes is probably due to Yule [17], but the use of preferential attachment in network models is due to Price [16]. Both Barabási-Albert (BA) and Price’s model also make use of ‘growth,’ where the whole network is constructed by adding nodes and links over time. However, initially these preferential attachment models did not focus on any other graph properties other than degree distribution. Later papers [18, 19] look at clustering coefficient along with degree distribution, but this is not as readily applicable in case of directed acyclic graphs (DAGs). Citation networks, and in our case scientific concept hierarchies, are directed, and the clustering coefficient for acyclic graphs is always zero. Unlike small world models for networks [20], preferential attachment models do not focus on graph diameters. However, Dommers et al. [21] proved a theoretical bound on diameters of scale-free networks. In most cases, it is assumed that the graph is undirected and fully connected. We found that the scientific concept hierarchies disconnected ‘tree-like’ structures. Little work is done to explain the graph properties other than the degree distribution of ‘tree-like’ graphs. We propose a generative model that looks at multiple graph-properties other than degree distribution: component size distribution and diameter of each connected component. Additionally, as we have time-annotated data, we can analyze the time difference between the ‘parent’ concept and the ‘child’ concept as an extra property. As Price’s model works with citation network (another DAG), we use this model as a starting point of our generative model.

There are many other models that use preferential attachment to explain scale-free networks. Dangalchev [22] proposed a two-level network model for collaboration networks that makes use of second order preferential attachment. There are models that explain power-law degree distribution without preferential attachment. ‘Copying models’ [23, 24] assume that nodes copy most of their links from a previously existing node. Hierarchical network models [25] also generate scale-free networks. It is worth noting that all of the models mentioned here employ the ‘growth’ mechanism for networks. However, there are generative models—such as the fitness model [26]—that produce scale-free networks without using growth. As the network discussed in the paper is built slowly over time, we restrict ourselves to generative models that utilize growth.

## Scientific concept hierarchies

In this study we make use of our previously generated scientific concept hierarchies. We briefly describe the creation and properties of the hierarchy but refer the interested reader to [5].

Our source data is the full text of the ACM digital library (ACMDL). As a proxy for scientific concepts, we utilize extracted keyphrases. In our context, keyphrases are the names assigned by authors to specific artifacts or concepts. For example, a researcher will create an algorithm (the *artifact* to which they can *refer*) and will often ‘name’ it (e.g., *Least-Squares Support Vector Machine*). In certain communities (CS in particular) such naming (or branding) is common. Even if the naming is not done directly by the author, future researchers citing the original work will often create a name (as is often the case with concepts named after their creators). Pressures to be unambiguous in reference often lead to the creation of new terminology (though clearly ‘branding’ activities of various sorts may also motivate this activity).

To build a list of seed concepts we make use of scientific abbreviations. In our work, we have found that invariably, a name of a concept (even single word names) will be abbreviated. We verify the idea that abbreviations can be used as a proxy of keyphrases, at least in the domain of computer science, via an experiment. We took the 85 unique keyphrases (in this case, article titles) listed in the Wikipedia entry for *List of Machine Learning Concepts* (ranging from well-known terms like *Support Vector Machines* to less known *Information fuzzy networks*) and successfully found abbreviations along with their expansions (e.g. *“Support Vector Machines (SVMs)”* or *“Information Fuzzy Networks (IFN)”*) on the Web (using Google) in all 85 cases. Though the use of abbreviations might be more prevalent in the Machine Learning literature, we believe this holds for other scientific domains as well.

To build this abbreviation dictionary, we use abbreviation extraction [27] to mine keyphrases from the ACMDL full-text. The expansions of these abbreviations become our working set of concepts. From these, we will search through the text to find the first appearance of the expanded keyphrase (though often this first use of the full keyphrase corresponds to its first abbreviated use).

We retain keyphrases which are abbreviated at least three times. Keyphrases are then composed into concept hierarchies by using string containment as a proxy for extension. We have found this to be a reliable approach [5] as researchers are often under twin pressures: (1) to signal the source and community to which the new work belongs (e.g., *‘Support Vector Machines (SVM)’* or *‘Byzantine Fault Tolerance (BFT)’*); and (2) signaling novelty or contribution (e.g., *‘Least-Squares Support Vector Machines (LSSVM)’* and *‘Practical Byzantine Fault Tolerance (PBFT)’*).

**Fig 1:**
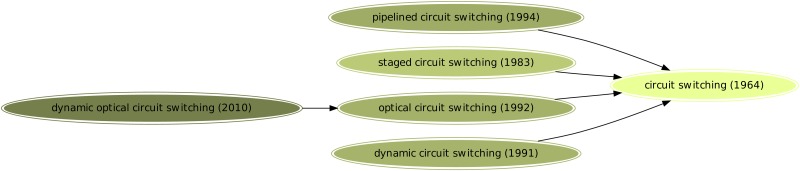
Keyphrase hierarchy for *Circuit Switching*. Earlier appearance is encoded with brighter color. The year of earliest appearance is shown along with the concepts.

**Fig 2:**
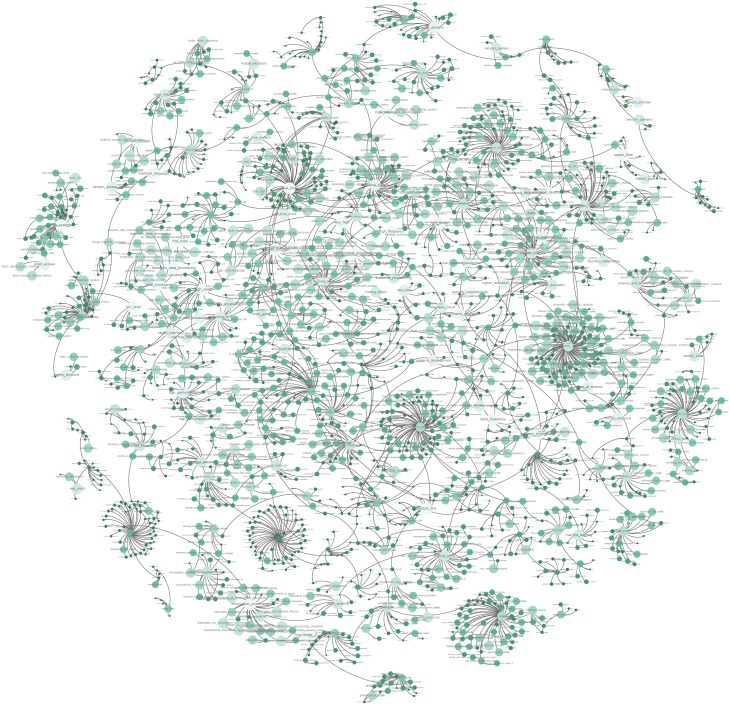
The giant component of our concept hierarchy. Nodes are colored (light to dark) and sized (large to small) based on the earliest concept.

Using this approach, we generated 1716 hierarchies which consist of 8661 unique key-phrases. Fig 1 depicts an example concept hierarchy for *Circuit Switching*. Most (1002, or 58%) are trees with only two nodes–one root concept and one child concept. The degree distribution of all hierarchies follows a power-law distribution with an exponent of -2.895. The size distribution of hierarchies also follows a power-law distribution with an exponent of -2.807. Surprisingly, there exists a giant component with 2302 nodes and 2436 edges (See Fig 2). The appearance of a giant component is explained by keyphrases derived from multiple other keyphrases. For example *Least-Squares Support Vector Machines (LS-SVMs)* has links to both *Least Squares* and *Support Vector Machines*. Thus multiple‘rooted’ trees are connected to form a single hierarchy. However, these keyphrases are relatively rare. Only 649 keyphrases are derived from more than one keyphrase with a majority being 2. Only 17 keyphrases are derived from three keyphrases. *Single Instruction Multiple Thread Evolution Strategy Pattern Search*, derived from *Evolution(ary) Strategy*, *Pattern Search*, and *Single-Instruction-Multiple-Thread* is a good example. These keyphrases may represent a conglomeration of different concepts or even different disciplines. However, this might also mean that the root keyphrases are very similar to each other, for example *likelihood ratio* and *log likelihood*. Our system currently does not interpret such ambiguity.

The existence of different power-law distributions (both for degree and size) is consistent with other phenomena. Power-law distributions are common in many different kinds of networks including citation networks. Preferential attachment models [13, 16] are often used to explain the power-law degree distribution of networks. However, it is not the only generative model to explain power-law distributions. For example, a power-law degree distribution can also be explained using vertex-copying models [23, 24].

Each connected component in the set of hierarchies can be represented as a collection of concepts in one discipline or sub-discipline. However, this criterion is very strict as we only consider string containment. The giant component consists of 26.57% of all nodes present in the set of hierarchies, and it connects 241 different root keyphrases including *data mining, web service, embedded system, semantic web, database management system, electrical engineering, user interaction, computational linguistics, human-computer interaction* and *information technology*.

### Interpreting the edges

Semantically, edges in a hierarchy can be most easily understood to mean Extends or IsSubtypeOf in most cases. To verify this, we chose 200 edges at random and manually validated this interpretation. We find that 92% (184) of those can be interpreted in this way. The remaining 16 cases are mostly due to normalization errors, For example “session identifier” and “session id.” are connected through an extension edge, though they are better handled as synonyms. We do not presently deal with negation in names (e.g., *non-monotonic reasoning* and *monotonic reasoning*). However, we find that only 0.6% of all edges include a negation in one of the terms (e.g. not or non-).

### Temporal properties

The edges derived only from string containment also represents a temporal pattern. For two concepts *A* and *B*, if *B* is derived from *A*, it is almost always the case that *B* is first mentioned after *A* in the scientific literature. Among 7136 edges, we found only 165 cases where the parent concept appears after the child concept. We believe that these are due to missing data (the parent concept appears outside ACM) or publication ordering (the gap between two concept appearing is just a year). This pattern can be explained by the tendency of the authors to extend previously known keyphrases. For each concept, we use the year the keyphrase was first found in the ACMDL. This is a limitation of the current work as we do not know if a term was used earlier, outside of the corpus. Additionally, if the keyphrase was created by a citing author (rather than the original), our algorithm may assign the wrong date. Qualitatively, we have found that this approach is largely accurate and have found only rare instances where the date was more than a year off from ground truth.

Due to the way we construct the trees, as the ‘depth’ of a keyphrase in a hierarchy increases, so does the length of the keyphrase. However, as described in [5], we also find that nodes at increasing depth are created later than those closer to the root. Thus, the average length of a keyphrase appears positively correlated with time. The relative change in length increases over time as new concepts are added. For example, we see *Petri Net (PN)* extended by *Queueing Petri Net (QPN)* which is extended by *Hierarchically Combined Queueing Petri Nets (HCQPN)*, which is finally extended by *Extended Hierarchically Combined Queueing Petri Nets (EHCQPN)*.

**Fig 3:**
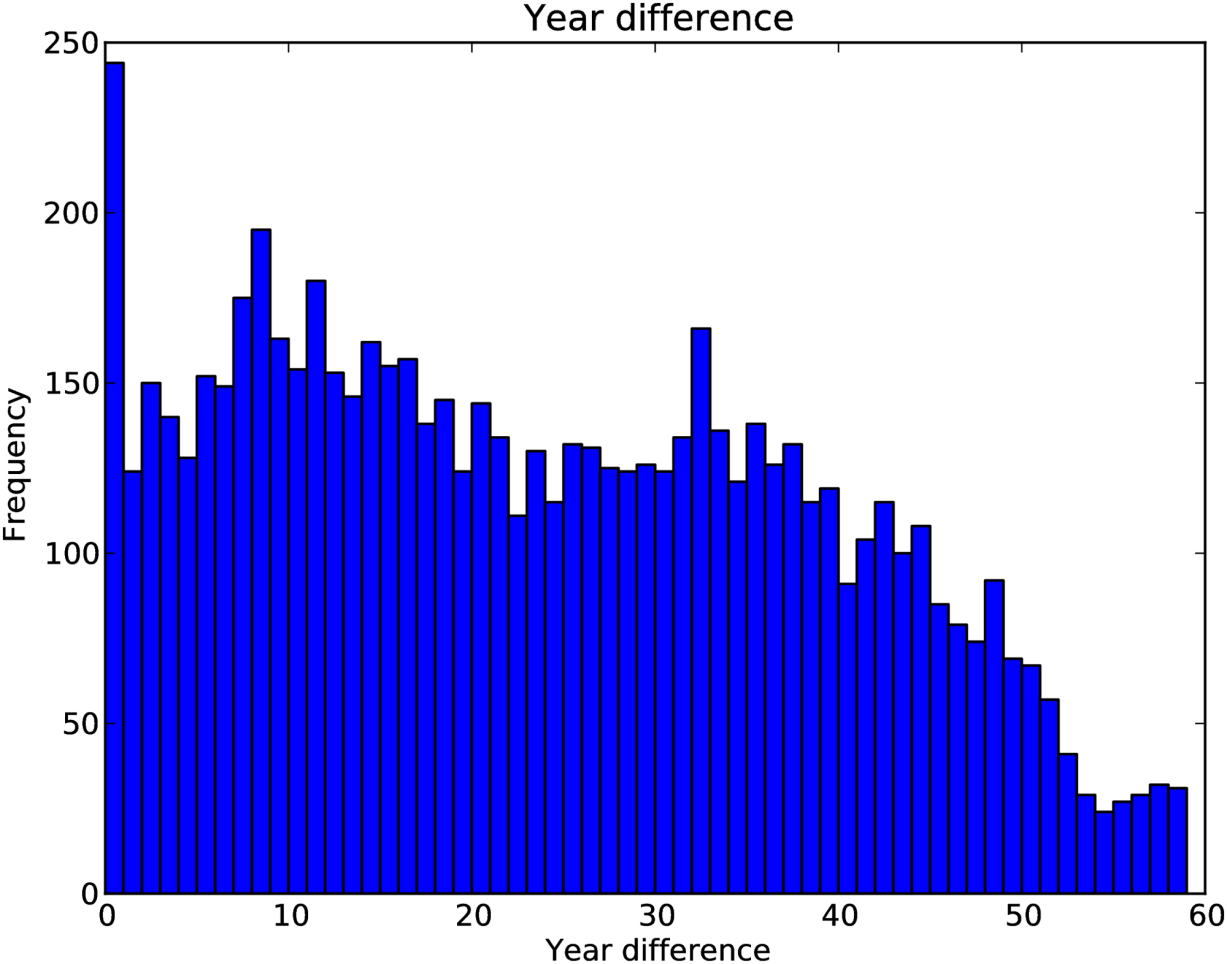
Histogram of year difference between first appearances of parent and child concepts.

Fig 3 depicts a histogram showing the year difference between first appearances of ‘parent’ and ‘child’ concepts. That is, after how many years a parent concept is extended by a child concept. We disregard the cases where the parent concept comes after the child concept as we believe these are due to the incompleteness of the data or publication ordering and the parent concept appearing outside ACM literature first. The plot shows a high frequency for year difference zero, which is again mostly due to publication ordering and in general we observe that ‘recent’ concepts are more likely to be extended. However, we do not assume that extending a concept depends only on how ‘recent’ it is.

### Other key statistics

**Fig 4:**
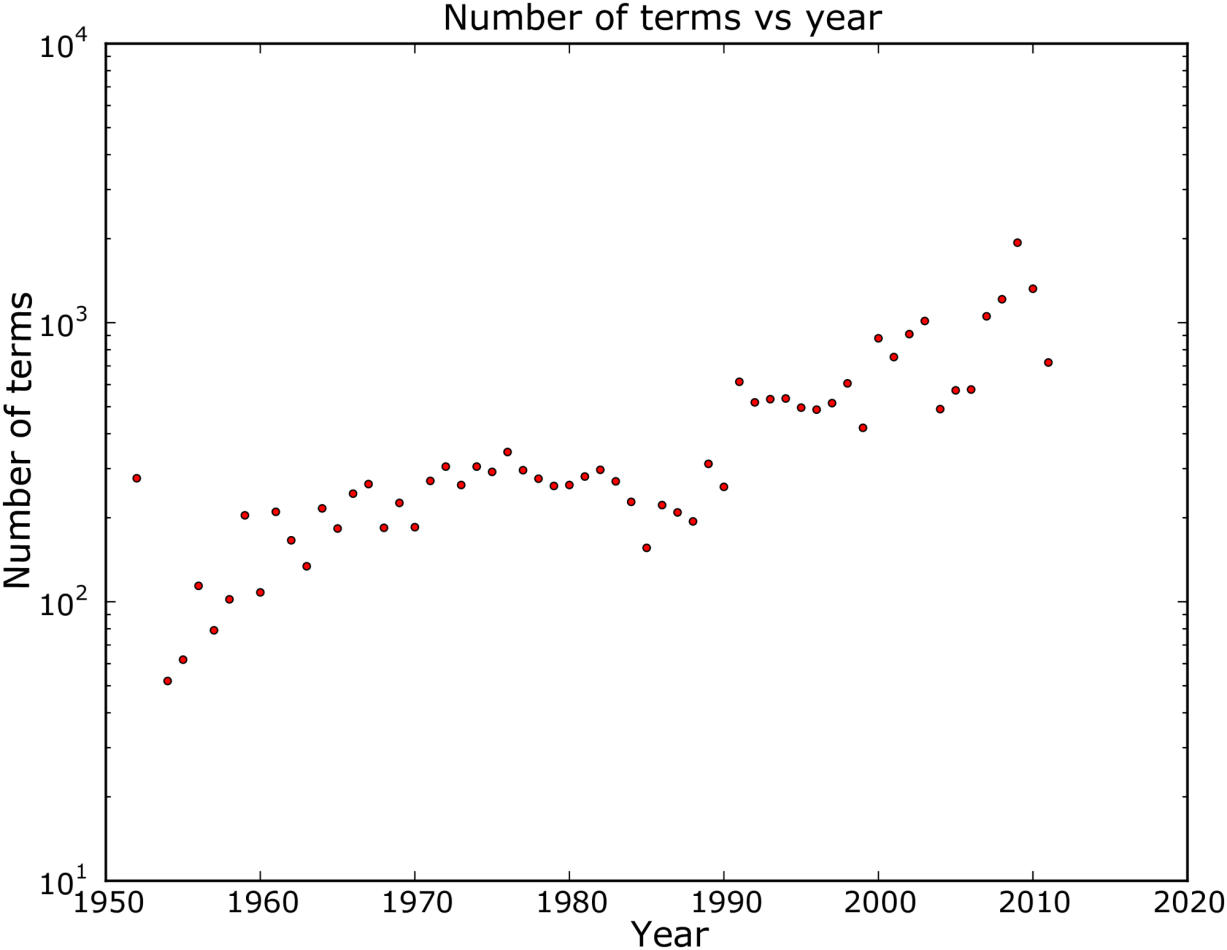
Number of new terms per year in ACMDL.

We also focus on the number of concepts per year in ACMDL corpus as it affects the keyphrase hierarchies. Fig 4 shows new concepts or terms per year in log-scale. This shows that the number of concepts increases more or less exponentially over the years (linear in log-scale). This indicates that most of the keyphrases in the hierarchies were created in recent years. The exponential increase of some concepts is largely due to the exponential increase of scientific papers published over the years.

**Fig 5:**
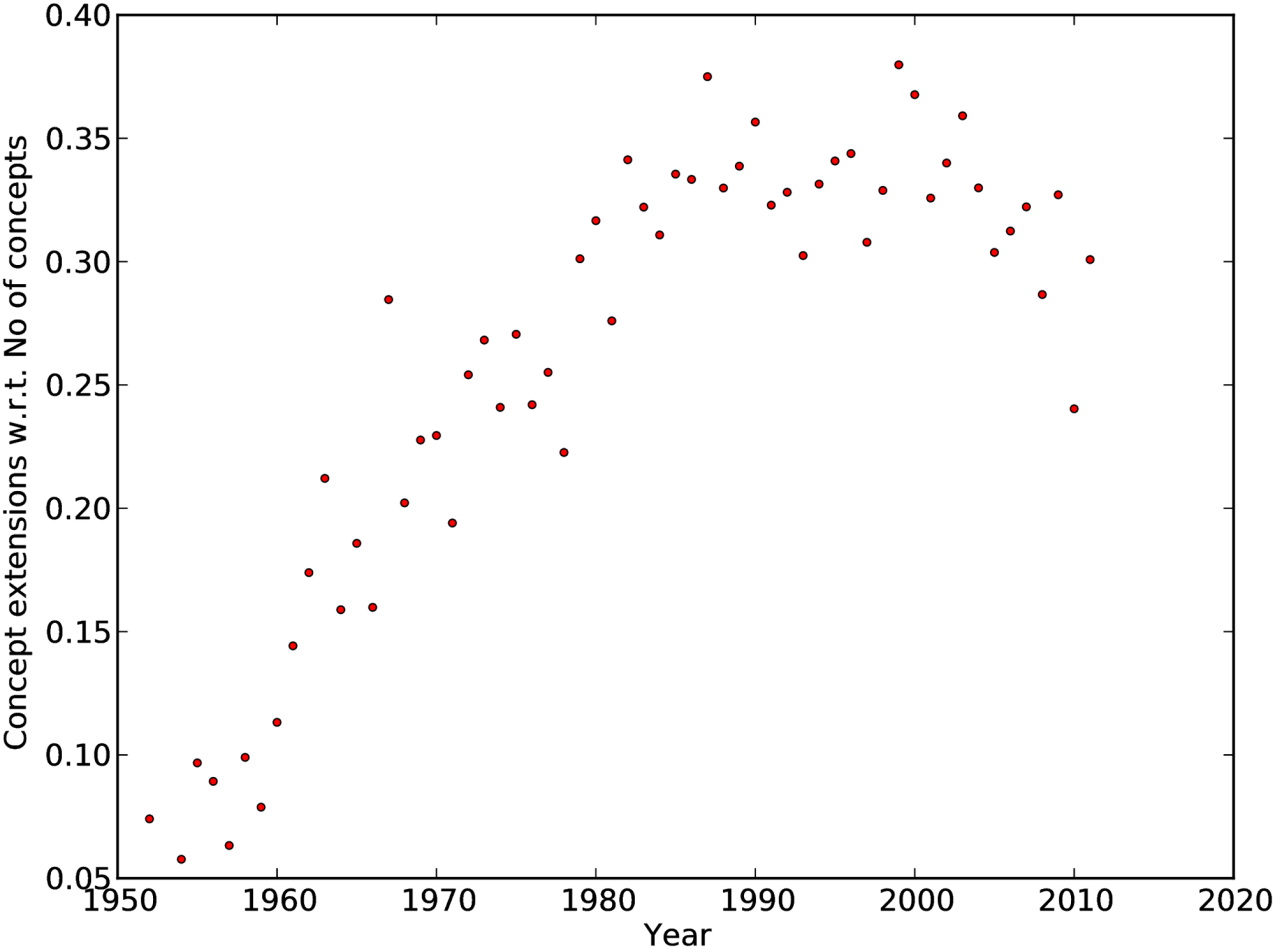
Concept extensions with respect to number of new concepts per year.

Notably, the number of derived concepts increase over time. Fig 5 shows concept extensions for each year. As the number of keyphrases also increase over time, we normalize the number of concept extensions in a year by the number of new concepts in that year. Note that we count all extensions of a concept in a *specific year*. For example, if concept *A* is extended five times in the year 1976, we count five extensions for *A* in 1976 (instead of one). We initially see an increasing tendency which seems to stabilize in 1990-2010. Our copy of the ACMDL does not contain the full set of computer science papers published in ACM after 2011 which may explain the decrease in concept extensions after that period.

## Generative model

To build a generative model for our concept hierarchies we begin by articulating the key metrics that the model should emulate. We then demonstrate how an extension of Price’s model leads to an appropriate model.

### Important graph properties

An important aspect of a graph-generation model is that it replicates certain properties of the original graph. In general, the most important of these properties are degree distribution of the graph, clustering coefficient, graph diameter and component size distribution (if the graph is not connected). However, recall that our concept hierarchies are a set of directed acyclic graphs (DAG). Thus, some assumptions on metrics do not hold. For example, the clustering coefficient would always be zero. Additionally, many preferential attachment models assume that the whole network is a single connected component and thus ignore modeling component size distribution. However, this is not the case with the concept hierarchies as we need to model the component size distribution.

We also model the diameter of each connected component, but we choose to do that in a slightly different and more informed way. We can build a set of trees rooted at the concepts that are not derived from any other concept using the hierarchies (e.g., *Support Vector*). The trees in a connected component have overlap with each other. Thus the diameter of a connected component do not represent how deep a concept extension goes (e.g. the tree *Support Vector* → *Support Vector Machine* → *Least-Squares Support Vector Machine* has a depth of two). This is especially true for the giant component which encompasses a large number of trees. Therefore we model the tree depth of a component as this is representative of the depth of concept extension.

As the scientific concept hierarchies are time-annotated (each concept is annotated with the year it first appeared in ACMDL), we also model the year difference between first appearances of ‘parent’ and ‘child’ concepts. In our generative model, we focus on four properties: degree distribution, component size distribution, tree depth and year difference between first appearances of parent and child concepts.

### General intuitions

A concept is more likely to be extended due to a number of factors: it is novel (it is a newly invented or ‘recent’ concept); it is popular; it is easy to extend; it is an active field of research. As with many other generative models, including Price’s and the Barabási-Albert(BA) model, we assume that the number of times that a concept has been previously extended is a proxy for the popularity/novelty of that concept. If we depict each concept as a node in a graph and add a link to the corresponding node each time a concept is extended, our assumption is a preferential attachment mechanism based on indegree of the nodes in the graph.

We have noted before that the hierarchies generated from ACMDL follow a power-law degree distribution. A graph is said to follow a power-law degree distribution, if for a value *k* above some threshold value *k*_*min*_, the fraction of nodes having degree *k*, denoted by *P*(*k*) is proportional to *k*^−*γ*^ for some positive constant *γ*, i.e.

$$\begin{array}{c} P\left(k\right)∼k^{-\gamma } \end{array}$$ (1)

For most real-world networks (e.g., citation networks, the World Wide Web, etc.), *γ* lies between 2 and 3. This is true for the scientific concept hierarchies as well, with a *γ* of 2.895 as per our estimation [5]. Preferential attachment models with growth are shown to produce power-law degree distribution [13, 16], so our assumption is plausible.

It is interesting to note that while degree and indegree distributions of the scientific concept hierarchies follow a power-law, this is not the case with the outdegree distribution. The outdegree of a node in the hierarchies is denoted by the number of ‘parents’ a node has (e.g., *Fault Tolerance(FT)* is a parent concept of *Byzantine Fault Tolerance(BFT)*), which is 0 or 1 in most the cases and very rarely exceed 2.

We use Price’s model as a starting point for our generative model. There are a number of reasons behind this. First, Price’s model is a preferential attachment model for citation networks, another directed acyclic graph as the scientific concept hierarchies, which produces power-law degree distribution. Second, this model assumes preferential attachment using indegree of the nodes in the graph, and the resulting network produces an outdegree distribution that does not follow power-law (in fact, outdegree for a node in Price’s model is constant), but the degree and the indegree distribution follows a power-law. A brief description of the original Price’s model is provided below.

### Preferential attachment: Price’s model

Price’s model starts with an initial set of nodes (usually 2 to 5 nodes) and assumes that the network is being built by adding a node and its corresponding links at discrete time-step. At each time-step, a node is added to the graph along with a constant number of edges (denoted by *c*) from the current node to previously existing nodes. For each (previously existing) node *i*, the probability that a new edge would be attached to it, is proportional to *q*_*i*_ + *a*, where *q*_*i*_ is the indegree of the node and *a* is a positive constant. Since, a new node must attach to a previously existing node, the correctly normalized probability is given by,

$$\begin{array}{c} \frac{q_{i}+a}{\sum_{i}\left(q_{i}+a\right)}=\frac{q_{i}+a}{n\bar{q}+na}=\frac{q_{i}+a}{n(c+a)} \end{array}$$ (2)

where, *n* is the total number of nodes in the already constructed graph and $\bar{q}$ denotes the average indegree. We know that, average indegree of a graph must be equal to average outdegree of the graph. As we are adding exactly *c* edges each time-stamp, for large *n* the average outdegree is *c* (discounting the initial set of nodes). However, we can rewrite the probability as,

$$\begin{array}{c} \frac{q_{i}+a}{n(c+a)}=\frac{c}{c+a}\frac{q_{i}}{nc}+\frac{a}{c+a}\frac{1}{n}=\phi \frac{q_{i}}{nc}+\left(1-\phi \right)\frac{1}{n} \end{array}$$ (3)

where $\phi =\frac{c}{c+a}$. This means that the probability that a new edge would attach to vertex or node *i*, can be modeled as choosing a vertex according to its indegree with probability *ϕ* or else choosing a random vertex.

Using these probabilities, it can be mathematically shown that the resulting network will follow power-law indegree distribution with exponent $-(2+\frac{a}{c})$ [16, 28].

### Extending Price’s model

Price’s model assumes a single connected component and constant outdegree for all nodes in the graph, which is not the case with our constructed hierarchies. Thus, we modify and extend Price’s model to better fit our data.

#### Modeling concept outdegree

The outdegree of a node in the simulated graph is determined by the number of concepts extended by the corresponding concept. Unlike Price’s model, each new concept does not always extend a previous concept. Additionally, there are concepts that extend multiple other concepts. However, the probability that a particular concept will be extended by a ‘current’ concept is very low (it is a rare event). When a new concept is added, the number of concepts to be extended by a single concept is determined by the sum of a large number of rare events (for a large number of concepts). The law of rare events [29] states that *“the total number of events will follow, approximately, the Poisson distribution if an event may occur in any of a large number of trials but the probability of occurrence in any given trial is small”*. Using this, we can model the number of concepts to be extended by a single concept, using a Poisson distribution.

We know that the proportion of derived concepts changes over time (as shown in Fig 5), so using a single Poisson distribution for all nodes does not make sense. Ideally, we should construct a different Poisson distribution at each time-step when a new concept is added, but this leaves no room for estimating the parameters for each Poisson distribution. Additionally, after a paper is published, it takes some time to be known among fellow researchers. Thus we assume that number of concepts extended by new concepts in a particular year follows the same Poisson distribution. As we only have the year of the first appearance for each concept, we can not estimate parameters for Poisson distributions over a smaller unit of time. For each year, the parameter (mean) of the Poisson distribution is obtained from the actual concept hierarchies using maximum likelihood estimation. For year *k*, the parameter λ_*k*_ for the Poisson distribution is given by,

$$\begin{array}{c} \lambda_{k}=\frac{e_{k}}{n_{k}} \end{array}$$ (4)

Where *e*_*k*_ denotes the number of concepts extended in the year *k* (if concept *A* is extended multiple times, we count all extensions) and *n*_*k*_ is the number of new concepts in the year *k*. In the simulated network, for each year we use the actual number of new concepts from the extracted scientific concept hierarchies.

Adding a different number of links at each time-step does not change the basic probability equation of Price’s model by much. In this case, the normalized probability that a new edge will attach to node *i* with indegree *q*_*i*_ is given by,

$$\begin{array}{c} \frac{q_{i}+a}{n(\bar{q}+a)}=\frac{\bar{q}}{\bar{q}+a}\frac{q_{i}}{n\bar{q}}+\frac{a}{\bar{q}+a}\frac{1}{n}=\phi^{\prime }\frac{q_{i}}{n\bar{q}}+\left(1-\phi^{\prime }\right)\frac{1}{n} \end{array}$$ (5)

where $\bar{q}$ is the average indegree, *a* is positive constant and $\phi^{\prime }=\frac{\bar{q}}{\bar{q}+a}$. The resulting network will still follow power-law indegree distribution with exponent $-(2+\frac{a}{\bar{q}})$ [28]. Note that, *ϕ*′ is dependent on average indegree $\bar{q}$. If we use separate indegree averages for each year, *ϕ*′ is a function of year *k*. However, if we average over all nodes over all years, *ϕ*′ is independent of *k*. For our simulated hierarchies, *ϕ*′ is independent of *k*.

#### Extending multiple concepts

The concept hierarchies contain concepts which extend multiple other concepts. In many cases, these concepts extend a concept that was previously extended multiple times. On the other hand, the number of extensions of the other concepts extended by these concepts seems to follow a uniform distribution. To account for this in our generative model, we choose the first extended concept according to indegree only and choose additional extended concepts randomly. Intuitively, this means that concepts extending multiple concepts tend to extend one popular concept along with other random concepts. This does not follow Price’s model. However, we adjust the value of *ϕ*′, i.e., the probability of choosing a node according to indegree in other cases, so that ‘on average’ the probability of choosing a node according to indegree remains the same. If for concept *v*, the number of concepts extended by *v* is denoted by *par*(*v*), the adjusted value of *ϕ*′ is given by,

$$\begin{array}{c} \phi_{adj}=\frac{\phi^{\prime }\times \sum_{v}par(v)-\sum_{v|par(v)>1}1}{\sum_{v}par(v)-\sum_{v|par(v)>1}par(v)} \end{array}$$ (6)

This ensures that, theoretically, the resulting degree distribution would have the same power-law exponent.

### Model description

We describe our complete generative model for scientific hierarchies in this section. As in the original Price’s model, we start with five initial set of nodes and then add nodes one by one.

**Outdegree estimation:** We start by estimating the outdegrees for each node as described in Section Modeling concept outdegree. For each year *k*, we count the number of new concepts *n*_*k*_ and estimate the Poisson distribution parameter λ_*k*_. Now we generate *n*_*k*_ samples from a Poisson distribution with parameter λ_*k*_ and assign them as outdegrees of *n*_*k*_ nodes. Each node is also time-annotated with year *k*.

**Probability estimation:** For each new link, we estimate the probability of choosing a node according to indegree *ϕ*′ (described in Section Preferential attachment: Price’s model) by equating the power-law exponent of the degree distribution actual hierarchies to $-(2+\frac{a}{\bar{q}})$ where $\phi^{\prime }=\frac{\bar{q}}{\bar{q}+a}$. We adjust the value of *ϕ*′ as described in Section Extending multiple concepts to account for the empirical observations.

**Preferential attachment:** We add nodes sorted by year to make sure that child concepts always come after their parent concepts. Note that, we do not look at the contents of the node in this simulation. For each node, if the outdegree of the node is zero, we just add the node to the graph, otherwise, if the outdegree is one, we add the node to the graph according to Price’s model (as described in Section Preferential attachment: Price’s model). For nodes with multiple parents, we add one link by choosing a node according to indegree and add other links by choosing random nodes.

### Results

By following the mathematical derivation of Price’s model [16, 28], we can similarly prove that for a very large number of nodes, our model would generate a network with power-law degree distribution. But as we have finite data and we are also interested in properties of the graph other than degree distribution, we resort to computer simulation.

To account for probabilistic selections in the model, we simulate the model 100 times to generate 100 networks and compare their properties with the actual network. As we have only one actual hierarchy, we risk overfitting. However as we have time-annotated data, we can remedy this in the following way. If we only consider concepts up to a certain year and build hierarchies, as long as we have sufficient number of nodes, they should follow similar properties as the complete hierarchies. We can generate hierarchies until year *k* from original hierarchies just by discarding concepts that came after year *k*. We can use this truncated hierarchies and compare their properties with simulated truncated hierarchies. Note that as our simulated network is also time-annotated, we can get the simulated truncated hierarchies just by truncating the simulated complete network. Intuitively, we can think of this as simulating until a particular year.

For our extracted scientific concept hierarchies, we started with 24273 concepts among which only 8741 concepts were placed in a hierarchy. When simulating we include all 24273 concepts, the ones that are not placed in a hierarchy are treated as a component with a single node. Note that we add a total of 24273 nodes after the initial five nodes to avoid throwing out five random nodes which may affect the model. Though this makes the total number of nodes in the simulated network to be 24278, it does not significantly affect its properties.

As stated before, we look at the degree distribution, component size distribution, tree depth and year difference between parent and child concepts. In all cases, we do these comparisons after removing single nodes without any edges from the networks as these nodes do not contribute to the estimation of the power-law exponent of the degree distribution, tree depth and year difference in any way.

**Fig 6:**
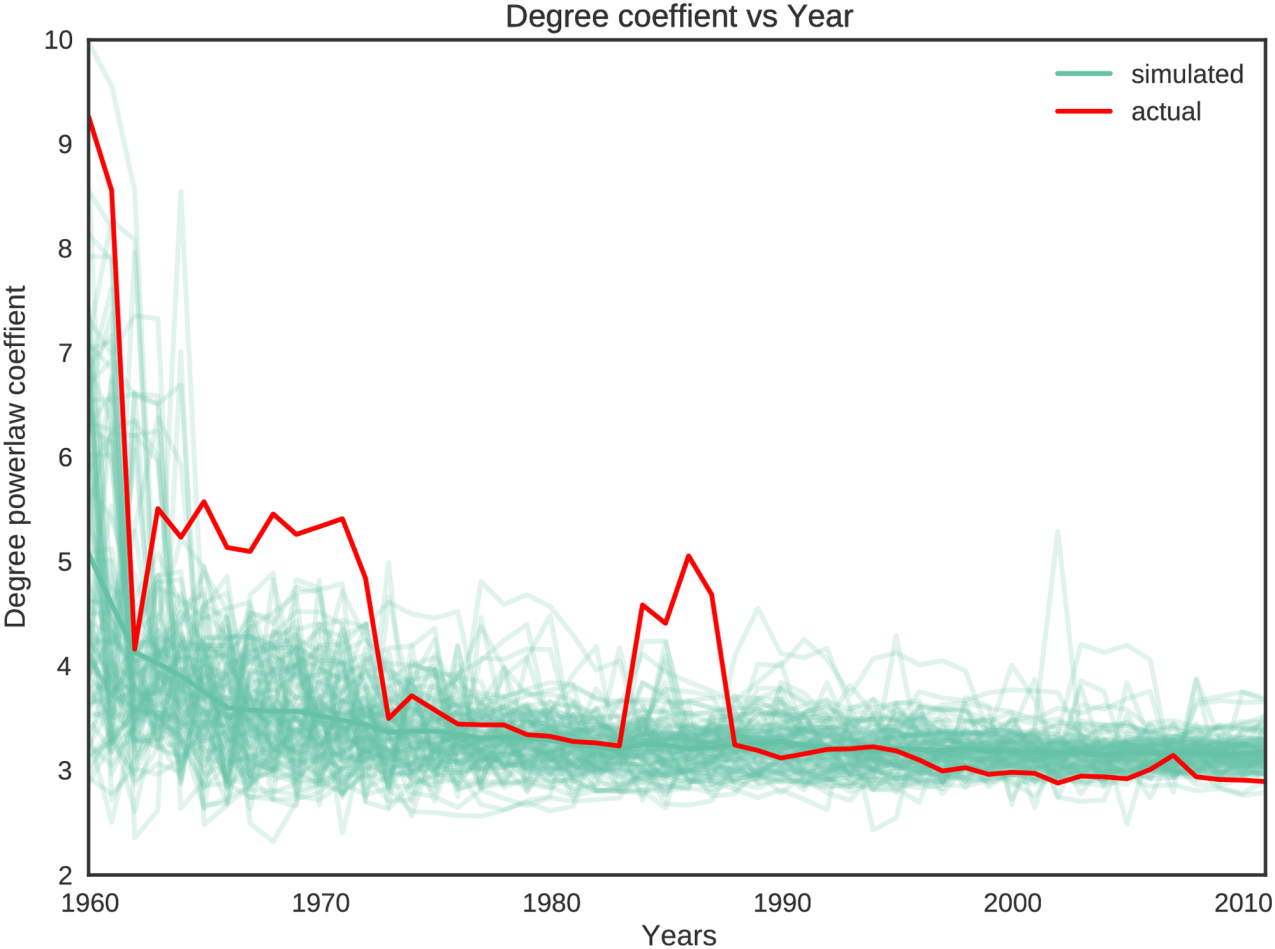
Comparison of power-law exponents (after negating) of degree distributions of actual and simulated networks over the years. The thin blue lines represent the exponent values for 100 simulated networks and the thick red line represent the exponents for the actual network.

**Degree distribution:** We compare the power-law exponents of the actual and the simulated networks from 1960 onwards. Fig 6 shows the comparison between power-law exponents for degree distributions of 100 simulated networks and the actual concept hierarchies. We ignore the negative sign of exponents in this and future plots. Initially, the number of nodes in the network is very low, and the result is noisy. Because of this we only show the comparison starting from the year 1960, after which we have a sufficient number of nodes in the network. We observe that in most cases, the power-law exponents of the simulated networks match very well with the actual network. Notably, after a certain threshold (in this case year 1987) the exponent does not vary much. Considering data from 1960 onwards, the correlation coefficient between the power-law exponents of the degree distribution of the actual hierarchies and the mean of the power-law exponents of the degree distribution of 100 simulated hierarchies is 0.886.

**Fig 7:**
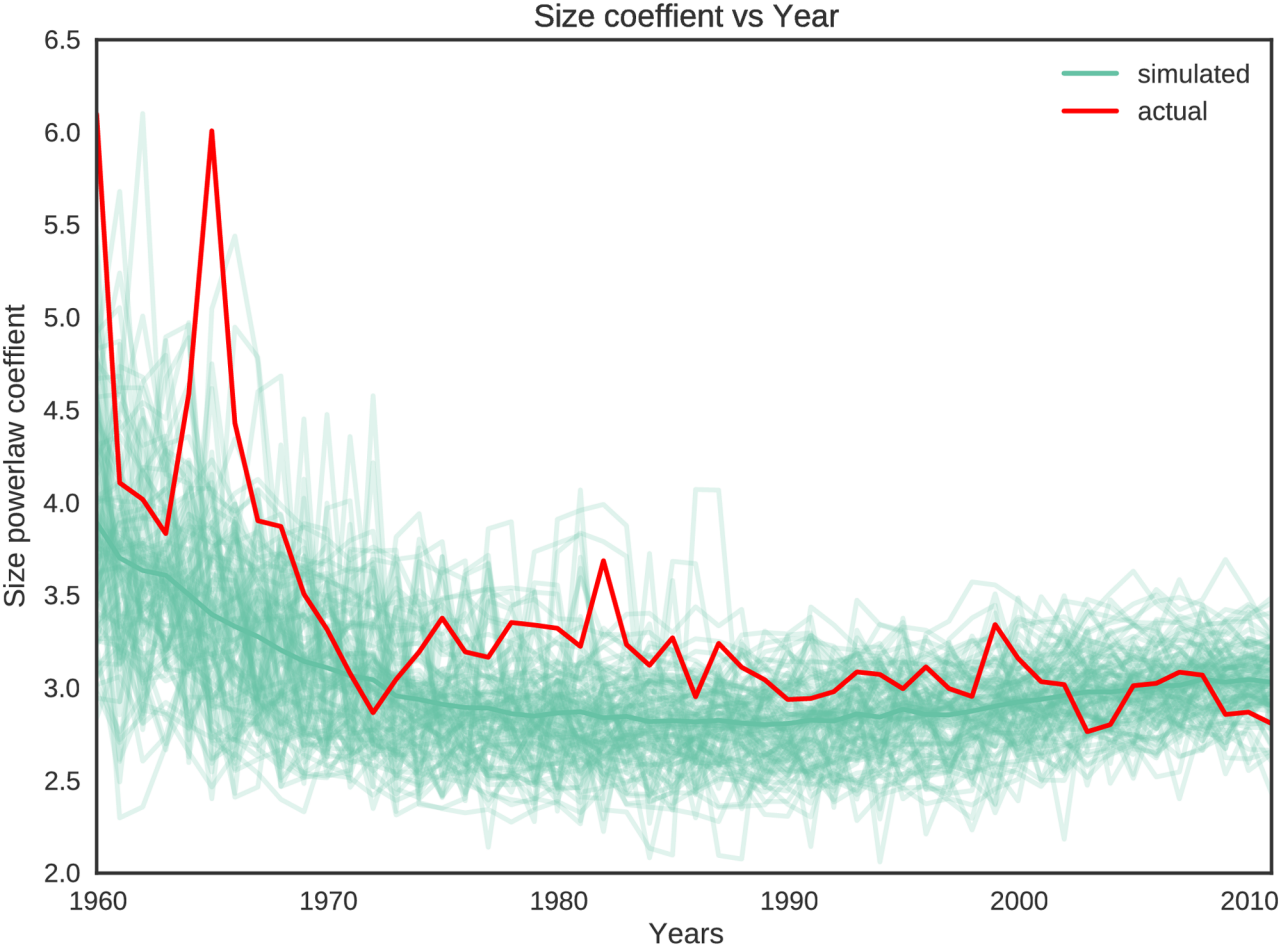
Comparison of power-law exponents (after negating) of component size distributions of actual and simulated networks over the years. The thin blue lines represent the exponent values for 100 simulated networks and the thick red line represent the exponents for the actual network.

**Component size distribution:** Similarly, we also compare the power-law exponents of the actual and the simulated networks from 1960 onwards. Fig 7 compares the power-law exponents of the component size distributions. As with degree distributions, we only show the comparison from the year 1960 when we have sufficient numbers of nodes in the network. Though somewhat noisy pre-1970 (where we do not have much data), the exponents for the actual hierarchies match well with those of the simulated hierarchies. The correlation coefficient between the power-law exponents of the component size distribution of the actual hierarchies and the mean of the power-law exponents of the component size distribution of the simulated hierarchies is 0.766. As with the degree distribution, we only use data from the year 1960 onward.

**Fig 8:**
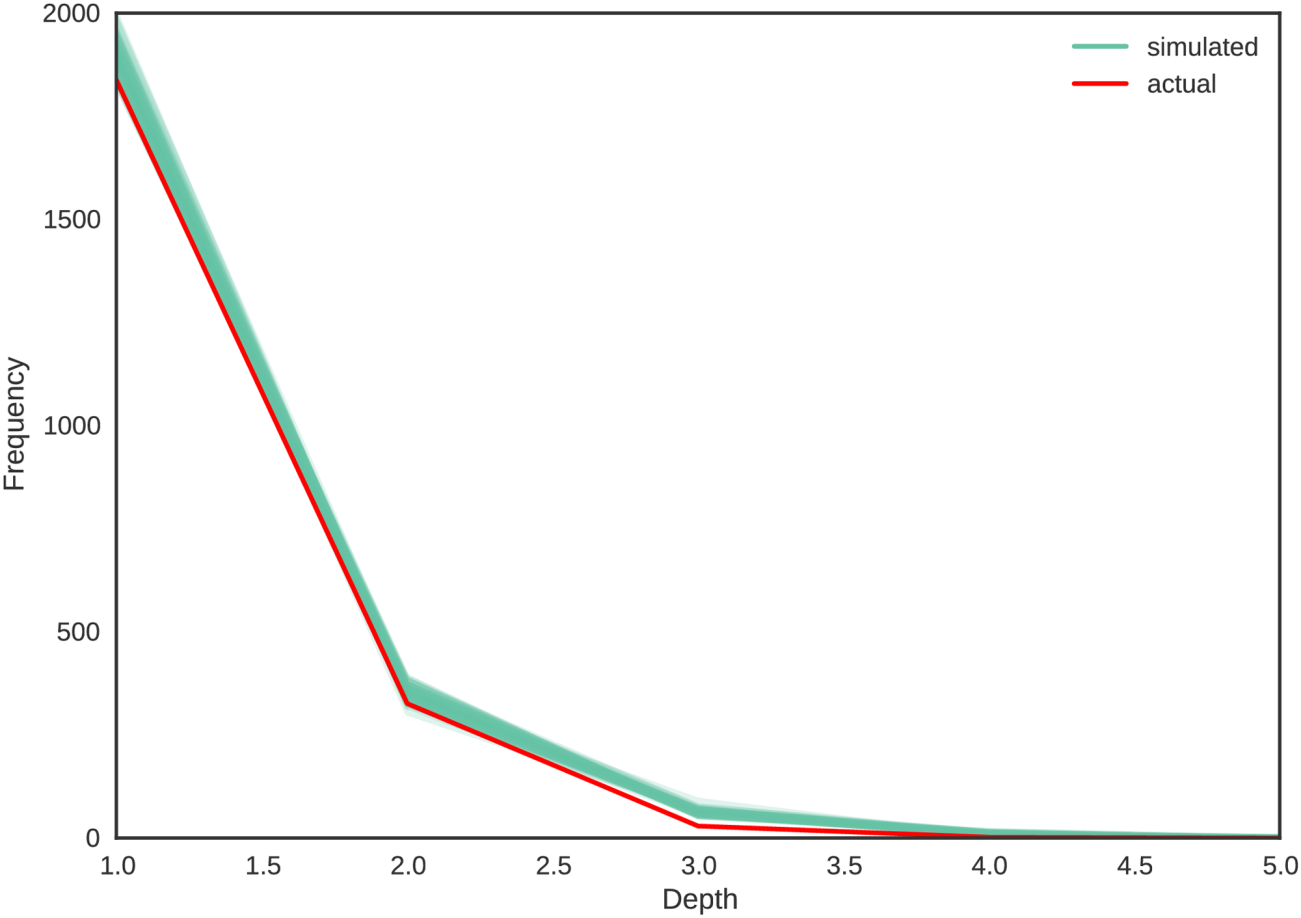
Comparison of tree depth distributions of actual and simulated networks. The thin blue lines represent the 100 simulated networks and the thick red line represent the actual network.

**Tree depths:** As stated before, we model tree depths (defined in Section Important graph properties) instead of diameters of connected components. Fig 8 depicts the tree depth distribution for simulated and actual networks. As we have five more nodes which are highly likely to be tree roots in simulated networks, the number of trees in simulated networks should be a little higher than the no of trees in the actual network. Considering this fact, the tree depths of the actual network matches closely with the simulated network. We do not use correlation coefficient to measure the goodness of fit as we only have tree depths from one to five.

**Fig 9:**
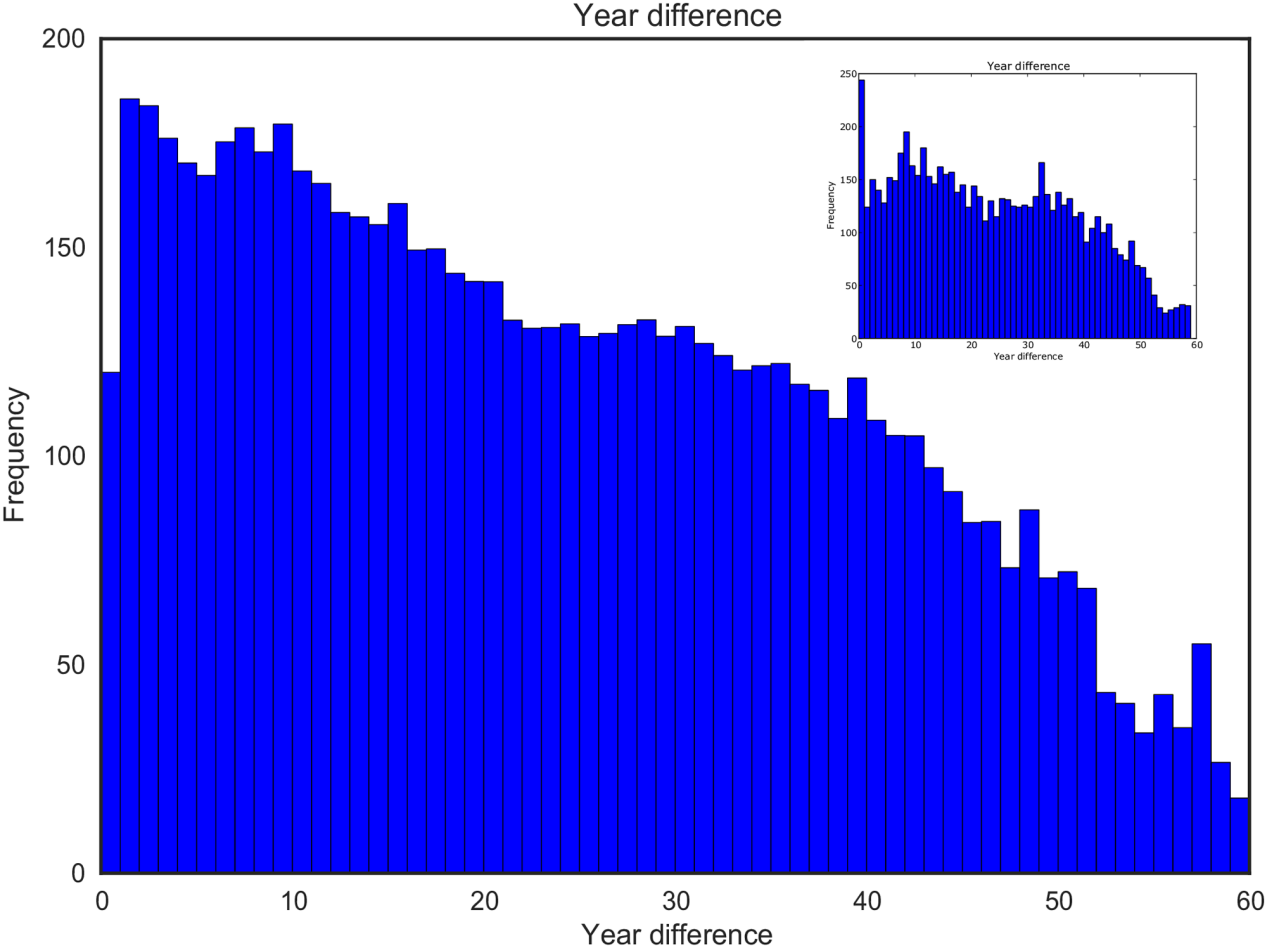
Histogram of year differences between child and parent concepts averaged over 100 simulated networks. Histogram of year differences between child and parent concepts for the actual network is shown in inset.

**Year difference between parent and child concepts:** Fig 9 illustrates the average year differences between first appearances of parent and child nodes in the 100 simulated networks, which is very similar to year differences between first appearances of parent and child concept in the actual scientific concept hierarchies (as shown in Fig 3 and the inset of Fig 9). The most notable difference between these figures is the frequency for year difference zero which is higher in case of the actual network. However, as we pointed out before this is most probably due to publication ordering or missing data in ACMDL. The correlation coefficient between the year difference of actual network and mean of the simulated networks is 0.875. However, if we disregard the cases where year difference is zero, the correlation coefficient becomes 0.934.

For all four sets of properties, we find that our generative model generates networks that are a close approximation to the actual concept hierarchies.

## Discussion and future work

There are a number of issues that may impact our results including how we interpret different types of concepts in our generative model. We briefly discuss some of these issues below.

The inclusion of nodes with zero edges (i.e., concepts that do not extend another concept and are not extended) while simulating the concept hierarchies, accounts for the fact that not all concepts are derived from other concepts and not every new concept is extended. Including these nodes also change the estimated probabilities which affected the simulated networks and their properties.

In our generative model, for each edge, the node it should attach to is chosen either according to indegree or randomly. At first glance, this seems to violate the assumption that ‘recent’ concepts are more likely to be extended. However, we still see that even in the simulated networks, year difference between first appearances of parent and child concepts is more likely to have a low value as shown in Fig 9. This apparently surprising result can be explained using the exponential growth in the number of concepts in recent years (as shown in Fig 4). As we have more concepts originated in recent years, if we choose concepts randomly we are likely to choose a modern concept. Concepts from recent years are *overall* ‘more important’ but a single concept from a recent year is not more important than an older concept. A recent study by Parolo et al. [30] shows a similar result for citations of scientific papers over the years.

As described above, it is important to note that we use the year of the first mention of a concept in the ACMDL as the ‘birth year’ of the concept, which is not necessarily true in many cases. In extreme cases, some of the concepts (especially if they are not from computer science) may have originated long before they are mentioned in the ACM digital library. We have begun to collect additional documents and ground truth annotations to better identify the concept birth year.

The concept hierarchies may enable search results that summarize key points in the development of an area (e.g., ‘Metro Maps of Science’ [31]). Understanding the evolution of scientific concepts has implications for both models of scientific development as well as in applied settings. For example, we have begun to leverage concept hierarchies to anticipate sub-fields that are growing rapidly. The model can also be used to predict future popularity of specific scientific concepts. Outside of computer science, the model can also be used in other scientific domains to compare and contrast scientific concept evolution in different domains.

## Conclusion

In this work, we describe our use of scientific keyphrase hierarchies to model concept evolution in the scientific literature. We study empirical properties of the concept hierarchies which includes graphical, temporal or semantic properties. We present an effective and intuitive graph generative model for these hierarchies. Along with diverse graph properties such as degree distribution, component size distribution and tree depth distribution, models temporal properties of the graph (year difference between parent and child concepts), which is unique compared to a majority of graph generative models.

## Supporting information

S1 File Scientific concept hierarchy dataset extracted from ACMDL. There is one file for every “tree” (note that some nodes will repeat between files). In each file there is 1 line per “edge.” The format is: child_concept, parent_concept, child_first_year, parent_first_year. The values are tab-separated. Spaces within concept names are replaced with an underscore. The first year corresponds to first year the concept was detected in the ACMDL corpus (note that this is generally correct, but can be off by a year or two).
(ZIP)
